# Unstable Object Points during Measurements—Deformation Analysis Based on Pseudo Epoch Approach

**DOI:** 10.3390/s22239030

**Published:** 2022-11-22

**Authors:** Robert Duchnowski, Patrycja Wyszkowska

**Affiliations:** Department of Geodesy, Institute of Geodesy and Civil Engineering, Faculty of Geoengineering, University of Warmia and Mazury in Olsztyn, 1 Oczapowskiego Street, 10-719 Olsztyn, Poland

**Keywords:** displacement analysis, pseudo epochs, M_split_ estimation, robust estimation, Monte Carlo simulations

## Abstract

Deformation analysis or point movement checking is the basis for monitoring ground or engineering structures. There are several approaches to conducting deformation analysis, which differ from each other in measurement techniques or data processing. Usually, they are based on geodetic observables conducted in at least two epochs. As such measurements are not “immediate”, it might so happen that a point (or some points) displaces during measurement within one epoch. The point movements might be continuous or sudden. This study focuses on the latter case, where rockburst, mining damages, or newly formed construction faults might cause displacement. To study this, an observation set consisting of measurements performed before and after point displacements is needed. As the actual observation division stays unknown, this can be called pseudo epochs. Such a hypothetical observation set requires special estimation methods. In this work, we examined M_split_ estimation and robust methods. The first approach’s advantage is that it provides two variants of the network point coordinates (before and after point movements), hence showing dynamic changes in the geodetic network. The presented empirical analyses confirm that M_split_ estimation is a better choice that results in better and more realistic outcomes.

## 1. Introduction

Monitoring dams, bridges, or other buildings and engineering structures is essential for their operation. Deformation or displacement analysis is often based on surveying measurements, built-in autonomic sensors, or a combination of these approaches [1]. Deformation analysis with geodetic methods is based on the displacement of object points being part of a geodetic network or measured by techniques providing mass measurements (such as terrestrial or airborne laser scanning). Here, the displacement of object points is determined between at least two moments of time; hence, the deformation analysis is conducted in two (or more) so-called measurement epochs [2,3,4,5,6]. However, classical geodetic observations, namely, measurements of angles, distances, height differences, or GNSS observables, are not “immediate” at all network points. Thus, it requires some time to perform measurements [7,8]. Generally, one assumes that the network points (reference and object ones) are stable (undisplaced) during the measurements of each epoch. The question then arises, what if any point (or some points) is not? It is evident that such displacements would affect the observation set, with some observations relating to the stage before displacements and other observations relating to the stage after displacements. As all observations belong to one epoch, we can call such subsets observations of the first or second pseudo epoch; however, in practice, we do not know the assignment of each observation to either of the pseudo epochs. This problem arose, for example, in [9]. In such cases, one sudden ground movement is caused by occurrences such as a rockburst or mining damages. Newly formed construction faults, such as wall cracks or fissures, may also cause this problem. Undoubtedly, the unrecognized division of measurements into pseudo epochs would also affect further computations, such as deformation analysis. This would indeed happen if one applied the usual approach based on the least squares method without any statistical tests or analyses detecting “outlying” observations (in the problem considered here, the observations of one of the pseudo epochs could be regarded as outliers). The issue of detecting (or dealing with) outliers in deformation analysis or separating them from network deformation has been addressed in several publications, e.g., [10,11,12,13]. Generally, in the context of pseudo epochs, one can apply a robust estimation method or other alternative methods that deal with an observation set consisting of the mentioned subsets.

In the first case, any robust M-estimation can be applied, e.g., the Huber method, the Hampel method, the Tukey method, the Danish method, or the IGG scheme [14,15,16,17,18,19,20,21]. The main difference between the mentioned robust M-estimation methods concerns their influence functions, which describe how the estimator responds to outliers. The other approach is R-estimation [10,22] based on rank tests. Two types of R-estimates are worth noting: R-estimate of the expected value and R-estimate of the shift between two samples. In the basic variants, these estimates are called the Hodges–Lehmann estimates (HL) [22]. Additionally, another variant of R-estimates, i.e., the Hodges–Lehmann weighted estimates (HLW) [10], can be used when observations have different accuracies.

An alternative approach might be the application of M_split_ estimation, which is a development of M-estimation. This method was created to estimate the location parameters (more generally, the parameters of the split functional models) when an observation set is an unknown mixture of realizations of different random variables. In such a case, a classical functional model is split into (at least) two competitive functional models. It is also noteworthy that before the estimation process, there is no a priori information on how the observation set is divided into the respective subsets. The assignment of each observation to either of the observation groups is carried out automatically during the iterative process. The novelty of such an approach is that competitive functional models also mean competitive versions of parameters and observation errors. Concerning the issue of point displacement between measurements in the same epoch, one competitive functional model can be assigned to each pseudo epoch; hence, point coordinates before and after point displacement can be estimated in one estimation process. The basic M_split_ estimation variant is called the squared M_split_ estimation (SMS) [23]. It can be derived under the assumption that observation errors are normally distributed. Another variant of M_split_ estimation is the absolute M_split_ estimation (AMS), which is based on the L_1_ norm condition [24,25]. The development of M_split_ estimation has led to some modifications of SMS or AMS methods [26,27,28,29,30], which have already been applied in deformation analysis and displacement detection [23,25,27,28,29,30,31,32,33,34].

In this work, we aimed to compare two approaches to the problem of the possible existence of pseudo epochs. The following methods were analyzed in the first approach: the least squares estimation (LS), the Huber method (robust M-estimation as example), and Hodges–Lehmann weighted estimation (R-estimation as example). In the second approach, we examined both the squared M_split_ estimation and the absolute M_split_ estimation variants.

## 2. Models, Foundations, and Algorithms of Methods Applied

Let us consider the following linear model that is often used for geodetic measurements:

(1) y=AX+v

where **y** is the observation vector n×1, **A** is the full rank coefficient matrix n×r, **X** is the parameter vector r×1, and **v** is the measurement error vector n×1. The LS estimator ${\hat{X}}_{LS}$ of the parameter vector can be computed as follows:

(2) $${\hat{X}}_{LS}={\left(A^{T}PA\right)}^{-1}A^{T}Py$$

where **P** is the diagonal weight matrix n×n. Here, we assume that the observations are independent; however, if they are not and the weight matrix is not diagonal, the solution remains the same. A similar solution computed in an iterative process concerns M-estimators. Here, we consider the Huber method, so the estimator ${\hat{X}}_{H}$ of the parameter vector is determined as follows [16,35,36]:

(3) $${\hat{X}}_{H}={\left(A^{T}WA\right)}^{-1}A^{T}Wy$$

(4) $${\left[W\right]}_{ii}={\left[P\right]}_{ii}\cdot w({\hat{v}}_{i})$$

where **W** is the diagonal matrix of weights n×n, $w({\hat{v}}_{i})$ is the weight function related to a variant of M-estimation and 1≤i≤n, ${\hat{v}}_{i}$ is the standardized error of *i*th observation, and ${\left[\circ \right]}_{ii}$ is the *i*th diagonal element of a matrix. The standardized error can be computed in the following way:

(5) $${\hat{v}}_{i}=\frac{v_{i}}{\sigma_{v_{i}}}$$

where $\sigma_{v_{i}}$ is the standard deviation of the error that can be computed by applying the approximation of the covariance matrix of errors in the following well-known form:

(6) $$C_{v}=\sigma_{0}^{2}\left(W^{-1}-A{\left(A^{T}WA\right)}^{-1}A^{T}\right)$$

where $\sigma_{0}^{2}$ is the variance of unit weight [37]. Another way to determine the error standard deviation is based on the application of Monte Carlo simulations [38]. The new version of the matrix **W** is computed in each subsequent iterative step based on the standardized errors from the previous iterative step (in the first iterative step W=P). Here, we use the Huber method, where the weight function is written in the following general formula [16,35,36]:

(7) $$w\left({\hat{v}}_{i}\right)=\{\begin{array}{lll} 1 & \mathrm{for} & \left|{\hat{v}}_{i}\right|\leq c\\ \frac{c}{\left|{\hat{v}}_{i}\right|} & \mathrm{for} & \left|{\hat{v}}_{i}\right|>c \end{array}$$

where *c* is the positive constant defining the interval of acceptable standardized measurement errors. We assume that c=2 in the numerical tests, which is often used in practice [17]. The constant *c* can also be computed using Monte Carlo simulations as the critical value related to the associated probability level [38].

Another robust estimator that can be applied in the context of this paper is the Hodges–Lehmann weighted estimator of the expected value ${\hat{E}}^{HLW}\left(X_{j}\right)$, where 1≤j≤r. The general formula proposed in [10] is as follows:

(8) $${\hat{E}}^{HLW}\left(X_{j}\right)=\mathrm{medw}\left(\frac{z_{k}+z_{l}}{2}\right)$$

where medw(∘) is the weighted median operator, and *z_k_* and *z_l_* are the elements of a sample of size *s* (1≤k≤s, 1≤l≤s). In the context of this paper, the sample is created separately for each point coordinate (e.g., horizontal *X* or *Y* or vertical *H*) and consists of the coordinates computed by applying the raw observations and the reference point coordinates in all possible independent ways [10]. For example, in the case of a leveling network, one can compute the object point height by adding the respective measurements to the height of the chosen reference point; in a horizontal network, this can be achieved using angular or linear intersections, resections, or just a polar method.

The last method considered here, M_split_ estimation, requires other assumptions and algorithms. Generally, the method in question is a development of M-estimation derived under the assumption that an observation set is an unrecognized mixture of the realization of two (or more) random variables that differ in location parameters [23]. Such an assumption leads to the split of the functional model from Equation (1) into two competitive ones [23,25]:

(9) $$y=AX+v\Rightarrow \{\begin{array}{c} y=AX_{\left(1\right)}+v_{\left(1\right)}\\ y=AX_{\left(2\right)}+v_{\left(2\right)} \end{array}$$

where **X**_(*m*)_ are versions of the parameter vector, and **v**_(*m*)_ are versions of the measurement error vector; m=1 or 2.

The main objective of M_split_ estimation is to assess parameter versions. It can be done by solving the optimization problem, which is equivalent to minimizing the objective function. The two M_split_ estimation variants considered here, i.e., the squared and absolute M_split_ estimation, can be written as follows:

(10) $$\begin{array}{c} \varphi_{\mathrm{SMS}}\left(X_{\left(1\right)},X_{\left(2\right)}\right)=\sum_{i=1}^{n}v_{i(1)}^{2}v_{i(2)}^{2}\\ \varphi_{\mathrm{AMS}}\left(X_{\left(1\right)},X_{\left(2\right)}\right)=\sum_{i=1}^{n}\left|v_{i(1)}^{}\right|\left|v_{i(2)}^{}\right| \end{array}$$

The competitive M_split_ estimates of the parameter vectors, namely, ${\hat{X}}_{\left(1\right)}$ and ${\hat{X}}_{\left(2\right)}$, are determined in the iterative process by applying the modified Newton method [23]. Two variants of M_split_ estimation require different computing algorithms, which stem from the differences in the objective functions and their derivatives [25]. SMS estimation uses the traditional iterative process given in the following form [24]:

(11) $$\begin{array}{c} X_{\left(1\right)}^{j}=X_{\left(1\right)}^{j-1}+dX_{\left(1\right)}^{j}=X_{\left(1\right)}^{j-1}-{\left[H_{\left(1\right)}\left(X_{\left(1\right)}^{j-1},X_{\left(2\right)}^{j-1}\right)\right]}^{-1}g_{\left(1\right)}\left(X_{\left(1\right)}^{j-1},X_{\left(2\right)}^{j-1}\right)\\ X_{\left(2\right)}^{j}=X_{\left(2\right)}^{j-1}+dX_{\left(2\right)}^{j}=X_{\left(2\right)}^{j-1}-{\left[H_{\left(2\right)}\left(X_{\left(1\right)}^{j},X_{\left(2\right)}^{j-1}\right)\right]}^{-1}g_{\left(2\right)}\left(X_{\left(1\right)}^{j},X_{\left(2\right)}^{j-1}\right) \end{array}$$

(12) $$\begin{array}{c} H_{\left(1\right)}\left(X_{\left(1\right)},X_{\left(2\right)}\right)=\frac{\partial^{2}\varphi \left(y;X_{\left(1\right)},X_{\left(2\right)}\right)}{\partial X_{\left(1\right)}\partial X_{\left(1\right)}^{T}}=2A^{T}w_{\left(1\right)}\left(v_{\left(1\right)}^{},v_{\left(2\right)}^{}\right)A\\ H_{\left(2\right)}\left(X_{\left(1\right)},X_{\left(2\right)}\right)=\frac{\partial^{2}\varphi \left(y;X_{\left(1\right)},X_{\left(2\right)}\right)}{\partial X_{\left(2\right)}\partial X_{\left(2\right)}^{T}}=2A^{T}w_{\left(2\right)}\left(v_{\left(1\right)}^{},v_{\left(2\right)}^{}\right)A \end{array}$$

(13) $$\begin{array}{c} g_{\left(1\right)}\left(X_{\left(1\right)},X_{\left(2\right)}\right)={\left[\frac{\partial \varphi \left(y;X_{\left(1\right)},X_{\left(2\right)}\right)}{\partial X_{\left(1\right)}}\right]}^{T}=-2A^{T}w_{\left(1\right)}\left(v_{\left(1\right)}^{},v_{\left(2\right)}^{}\right)v_{\left(1\right)}^{}\\ g_{\left(2\right)}\left(X_{\left(1\right)},X_{\left(2\right)}\right)={\left[\frac{\partial \varphi \left(y;X_{\left(1\right)},X_{\left(2\right)}\right)}{\partial X_{\left(2\right)}}\right]}^{T}=-2A^{T}w_{\left(2\right)}\left(v_{\left(1\right)}^{},v_{\left(2\right)}^{}\right)v_{\left(2\right)}^{} \end{array}$$

(14) $$\begin{array}{c} w_{\left(1\right)}\left(v_{\left(1\right)}^{},v_{\left(2\right)}^{}\right)=\mathrm{diag}\left[w_{\left(1\right)}\left(v_{1(1)}^{},v_{1(2)}^{}\right),\ldots ,w_{\left(1\right)}\left(v_{n(1)}^{},v_{n(2)}^{}\right)\right]\\ w_{\left(2\right)}\left(v_{\left(1\right)}^{},v_{\left(2\right)}^{}\right)=\mathrm{diag}\left[w_{\left(2\right)}\left(v_{1(1)}^{},v_{1(2)}^{}\right),\ldots ,w_{\left(2\right)}\left(v_{n(1)}^{},v_{n(2)}^{}\right)\right] \end{array}$$

where $dX_{\left(m\right)}^{}$ are increments to the parameter vector, $H_{\left(m\right)}\left(X_{\left(1\right)},X_{\left(2\right)}\right)$ are Hessians, $g_{\left(m\right)}\left(X_{\left(1\right)},X_{\left(2\right)}\right)$ are gradients, $w_{\left(m\right)}\left(v_{\left(1\right)}^{},v_{\left(2\right)}^{}\right)$ are matrices of the weight, diag(∘) is the diagonal matrix; m=1 or 2. The necessary weight functions of SMS estimation are as follows [23]:

(15) $$\left\{ \begin{array}{l} w_{\left(1\right)}\left(v_{i(1)},v_{i(2)}\right)=v_{i(2)}^{2}\\ w_{\left(2\right)}\left(v_{i(1)},v_{i(2)}\right)=v_{i(1)}^{2} \end{array}\right.$$

The iterative process ends for the iterative step *t* for which both $g_{\left(1\right)}\left(X_{\left(1\right)}^{t-1},X_{\left(2\right)}^{t-1}\right)=0$ and $g_{\left(2\right)}\left(X_{\left(1\right)}^{t-1},X_{\left(2\right)}^{t-1}\right)=0$ or at least they are close enough to **0**, and the parameter changes between the subsequent iterative steps are smaller than the assumed tolerance [24,25]. Hence, finally ${\hat{X}}_{\left(1\right)}=X_{\left(1\right)}^{t}=X_{\left(1\right)}^{t-1}$ and ${\hat{X}}_{\left(2\right)}=X_{\left(2\right)}^{t}=X_{\left(2\right)}^{t-1}$ [23,25].

AMS estimation requires a parallel iterative process:

(16) $$\begin{array}{c} X_{\left(1\right)}^{j}=X_{\left(1\right)}^{j-1}+dX_{\left(1\right)}^{j}=X_{\left(1\right)}^{j-1}-{\left[H_{\left(1\right)}\left(X_{\left(1\right)}^{j-1},X_{\left(2\right)}^{j-1}\right)\right]}^{-1}g_{\left(1\right)}\left(X_{\left(1\right)}^{j-1},X_{\left(2\right)}^{j-1}\right)\\ X_{\left(2\right)}^{j}=X_{\left(2\right)}^{j-1}+dX_{\left(2\right)}^{j}=X_{\left(2\right)}^{j-1}-{\left[H_{\left(2\right)}\left(X_{\left(1\right)}^{j-1},X_{\left(2\right)}^{j-1}\right)\right]}^{-1}g_{\left(2\right)}\left(X_{\left(1\right)}^{j-1},X_{\left(2\right)}^{j-1}\right) \end{array}$$

The way of computing the Hessians and gradients is similar to Equations (12)–(14); however, following the objective function of AMS estimation presented in Equation (10), one should use the weight functions in the following forms [25]:

(17) $$\left\{ \begin{array}{l} w_{\left(1\right)}\left(v_{i(1)},v_{i(2)}\right)=\{\begin{array}{cc} -\frac{\left|v_{i(2)}\right|}{2v_{i(1)}} & \mathrm{for}\;v_{i(1)}<0\\ \frac{\left|v_{i(2)}\right|}{2v_{i(1)}} & \mathrm{for}\;v_{i(1)}>0 \end{array}\\ w_{\left(2\right)}\left(v_{i(1)},v_{i(2)}\right)=\{\begin{array}{cc} -\frac{\left|v_{i(1)}\right|}{2v_{i(2)}} & \mathrm{for}\;v_{i(2)}<0\\ \frac{\left|v_{i(1)}\right|}{2v_{i(2)}} & \mathrm{for}\;v_{i(2)}>0 \end{array} \end{array}\right.$$

The conditions for ending the parallel process are the same as in the traditional one. The differences between the presented algorithms concern the starting points and computing the estimates in subsequent iterative steps (compare Equations (11) and (16)). There is one starting point in the traditional iterative process (usually LS estimates of the parameters) and two different starting points in the parallel iterative process. A detailed description of the M_split_ estimation variants and algorithms and the relationship between their objective, influence, and weight functions can be found in [23,25].

Considering the problem of pseudo epochs, the competitive versions of the parameter vector should reflect the point displacements. One should expect one version to correspond to the point coordinates before displacements and the second version to after the point movements. Such a “double” solution is different than in the robust M-estimation, where the only solution is supposed to be free of the negative influence of “outliers” resulting from unexpected point displacements.

## 3. Empirical Tests

### 3.1. Simulated Leveling Network

**Figure 1:**
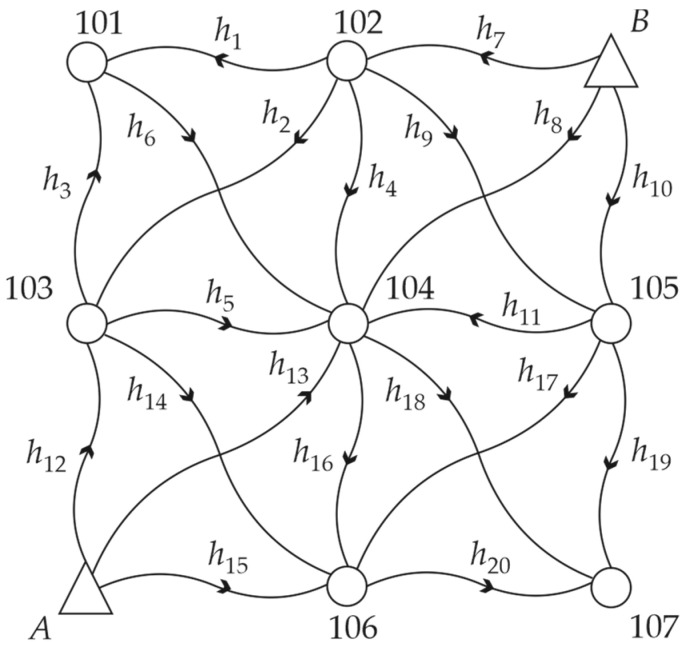
Simulated leveling network.

The first numerical test concerned a leveling network presented in Figure 1. This network included two reference points *A* and *B* and seven object points 101, 102, 103, 104, 105, 106, and 107 (points with unknown heights). We let 20 height differences *h_i_* be measured with the standard deviation of 1 mm twice in one measurement epoch; hence, we had 40 observations. Without loss of generality, one can assume that the theoretical heights of all network points equal 0 mm; therefore, theoretical height differences between every point pair also equal 0 mm.

To examine the two approaches presented thoroughly, we considered several variants of point displacements. We assumed that only points 101 and 102 were unstable in all variants. We took the values of $\Delta H_{101}=10\;\mathrm{mm}$ and $\Delta H_{102}=20\;\mathrm{mm}$ in Variants I and II, which differed from each other in the number of measurements performed after the displacements of the points. In Variant I, the second measurements of the height differences *h*_1_, *h*_3_, and *h*_7_ were measured after the point displacements; in Variant II, the first measurements of the height differences *h*_3_, *h*_7,_ and the second measurements of the height differences *h*_1_, *h*_3_, *h*_4_, *h*_7_, and *h*_9_ were measured after the point displacements.

In the classical approach, the observations measured after the point displacements should be regarded as outliers, contrary to M_split_ estimation, where such a subset should be related to the second competitive functional model. One should realize that in practice, we do not know which points are displaced and which observations are performed before and after the point displacements; thus, we cannot divide observations into subsets a priori.

We examined how such an affected observation set influenced the estimation results by applying crude Monte Carlo method. Hence, we assumed that the observations were normally distributed and simulated 5000 observation sets in each variant in Mathcad 15.0. Such a number of simulations guarantees the error is approximately 0.56% in crude Monte Carlo simulations, e.g., [39], which seems sufficient in the context of this paper. We examined how the methods mentioned previously, namely, LS estimation, the Huber method with the steering parameter c=2, HLW estimation, and two variants of M_split_ estimation (SMS and AMS methods), dealt with the problem of pseudo epochs. Because the accuracy of all observations was the same, we assumed that the weight matrix **P** was equal to the identity matrix **I**. The starting vector $X_{\left(1\right)}^{0}$ of the iterative processes of M_split_ estimation of Equations (11) and (16) was based on the heights of the reference points and the theoretical height differences; hence, its elements were equal to 0 mm. The starting vector $X_{\left(2\right)}^{0}$ (for AMS estimation) was computed by adding 10 mm to each element of the vector $X_{\left(1\right)}^{0}$ [24].

**Figure 2:**
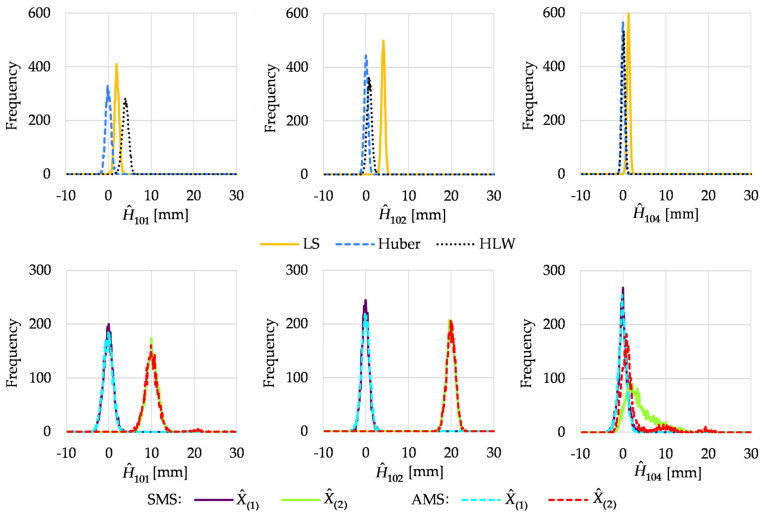
Histograms of estimated point heights in Variant I.

The histograms of the estimated heights of the chosen network points obtained in Variant I are presented in Figure 2. The histograms were determined for the heights of the object points that moved, namely, 101 and 102, and one chosen stable point, namely, 104. The histograms obtained for the other stable points were very similar to the histogram of point 104 height; hence, they are omitted here.

In Variant I, the best results were obtained for the Huber method, where the height estimates were robust against outlying observations. LS estimation was not robust; hence, its results were not acceptable, especially for unstable points. The other robust method (HLW) also did not provide correct results, especially for point 101. This was due to the location of outliers and the low breakdown point of the method in the case of a small number of observations (independently computed point heights in this case) [11]. Note that we had 6 independent ways to compute the heights of point 101, 8 ways for point 102, and 12 ways for point 104, which determines the size of the samples in Equation (8).

Following the split functional model of Equation (9), we had two competitive solutions in M_split_ estimation, namely, the competitive point height estimates reflecting two pseudo epochs. The histograms obtained for the estimated heights of unstable points showed that M_split_ estimation detected the point displacements correctly (the histograms coincided with all simulated point heights). The situation was different for the estimated height of point 104 (similar histograms were obtained for the rest of the stable object points). The first solutions of both variants seemed correct; the histogram coincided with 0 mm. The histograms of the second solutions were generally proper; however, they seemed slightly skewed to the right, especially in the case of SMS estimation.

**Figure 3:**
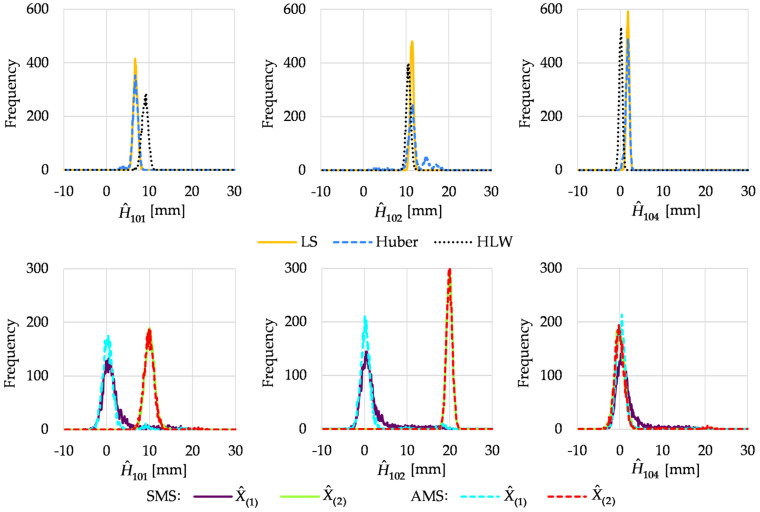
Histograms of estimated point heights in Variant II.

Histograms of the estimated point heights obtained in Variant II are presented in Figure 3. 

The conventional methods failed to estimate the heights of the unstable points. Only estimates of the heights of point 104 were close to the theoretical value (the best results were obtained for HLW estimation). The histograms obtained for the point heights estimated by M_split_ estimation were generally located correctly. However, the histogram of the first solution of AMS estimation obtained for point 101 was bimodal, with a very small second mode located at the other solution. This time, the histograms obtained for point 104 seemed less skewed to the right than in Variant I. It followed the general feature of M_split_ estimation, where it is easier to distinguish two groups of observations when there is a smaller discrepancy in the number of observations in the groups in question.

In the first two variants, the displacements of points 101 and 102 were relatively high. Next, we considered the simulated displacements whose values were at the same magnitude as the measurement accuracy. Thus, we considered Variants III and IV for which $\Delta H_{101}=3\;\mathrm{mm}$ and $\Delta H_{102}=2\;\mathrm{mm}$. Similarly to Variants I and II, we assumed that the second measurements of the height differences *h*_1_, *h*_3_, and *h*_7_ were measured after the point displacements in Variant III. In contrast, in Variant IV, the first measurements of the height differences *h*_3_ and *h*_7_ and the second measurements of the height differences *h*_1_, *h*_3_, *h*_4_, *h*_7_, and *h*_9_ were measured after the point displacements.

**Figure 4:**
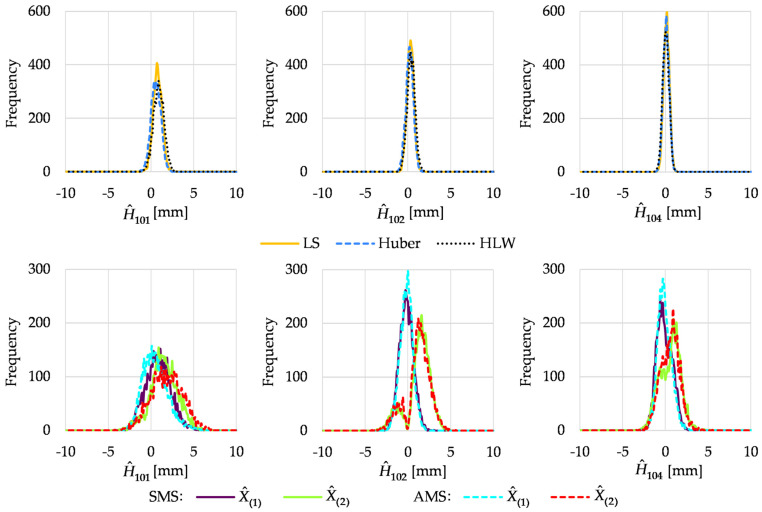
Histograms of estimated point heights in Variant III.

Figure 4 presents the histograms obtained in Variant III. 

The results of all conventional methods were very similar, resulting from small values of outliers (observations performed after the point displacements); hence, the robust methods did not consider them to be outlying. Only solutions obtained for point 104 were taken as correct, and the solution for point 102 was very close to the true value. For M_split_ estimation, the histograms for the estimated height of point 101 were broad; however, the maximum frequencies seemed to coincide with simulated point heights. In the case of point 102, the first solutions of SMS and AMS estimation were correct. The histograms of the second solutions were bimodal, with a minor mode located at the wrong solution. In the case of histograms of point 104, the first solutions of both variants seemed correct but the second solutions were not. This was due to the low observations performed after the point displacements.

**Figure 5:**
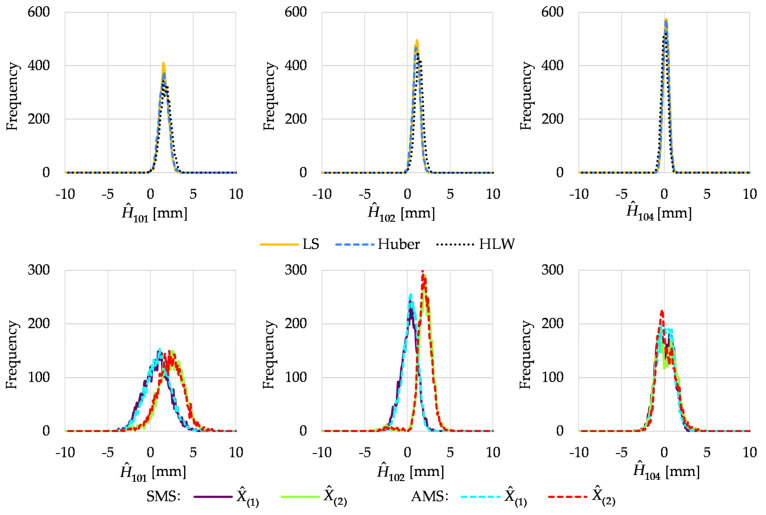
Histograms of estimated point heights in Variant IV.

The histograms of the estimated point heights in Variant IV are presented in Figure 5. The results of all conventional methods were very similar to each other. However, none of the methods provided satisfactory results. Only the histograms obtained for the height of point 104 were located close to 0 mm. This was due to the fact that most of the height differences between that point and the other network points were measured at the first pseudo epoch. The histograms obtained for M_split_ estimates of the heights of points 101 and 102 showed that M_split_ estimation detected the point displacements better than in the preceding variant. This was due to the higher number of observations in the second pseudo epoch, which helped to correctly separate the pseudo epochs. The histograms obtained for point 104 had modes close to 0 mm for both M_split_ estimation variants and competitive solutions.

The presented histograms gave only general information about estimation results. The crude Monte Carlo simulations allowed us to compute some descriptive parameters. We applied the median and the root-mean-square deviation (RMSD). The median here describes the central tendencies of the estimates. The mean values of estimates were omitted. They are usually equal to the respective medians or differ from them by no more than 0.2 mm.

On the other hand, RMSD describes the accuracy of the estimates, and it can be computed in the following way [25,40]:

(18) $$\mathrm{RMSD}=\sqrt{\sum_{i=1}^{5000}\frac{{\left({\hat{X}}_{i}^{MC}-X\right)}^{2}}{5000}}$$

where: ${\hat{X}}_{i}^{MC}$ is the estimated parameter in the *i*th simulation, and *X* is the simulated parameter. For the conventional method, for each point, X=0 mm. In the case of M_split_ estimation, X=0 mm for the first solution $X_{\left(1\right)}$. However, for the second solution $X_{\left(2\right)}$, $X=\Delta H_{101}=10\;\mathrm{mm},$ $X=\Delta H_{102}=20\;\mathrm{mm}$, or $X=\Delta H_{104}=0\;\mathrm{mm}$ in Variants I and II and $X=\Delta H_{101}=3\;\mathrm{mm},$ $X=\Delta H_{102}=2\;\mathrm{mm}$, or $X=\Delta H_{104}=0\;\mathrm{mm}$ in Variants III and IV.

Table 1 presents the medians of the estimated point heights, whereas Table 2 shows the RMSDs of the estimates. In Variant I, the tables confirm that the Huber method provided the correct results and LS or HLW estimation did not. They also prove that M_split_ estimation might be able to distinguish between the first and second pseudo epochs. What is more, it could assess the point displacements correctly. However, M_split_ estimation provided worse assessments of $X_{\left(2\right)}$ for the points that were not displaced during the measurements (see the results obtained in the case of point 104, especially RMSDs). This might be due to the small number of observations performed after the point displacements.

In Variant II, the best results, i.e., the medians closest to the theoretical values, were obtained for AMS estimation. Empirical accuracies for Variant II showed that M_split_ estimation could deal with the existing pseudo epochs better than the conventional methods when the number of observations carried out after the point displacements was more significant. Even the Huber method, which succeeded in Variant I, could not manage a higher number of outlying observations.

In Variant III, the outcomes obtained were worse. M_split_ estimation variants had a problem correctly distinguishing between the two pseudo epochs. When the displacement during measurements was small and close to 0 mm, the classical robust methods and LS estimation gave better results than the M_split_ estimation variants. However, it is hard to consider them as satisfactory in the example given (mainly results obtained for the unstable points).

In Variant IV, the median values obtained pointed out that the M_split_ estimation variants characterized the actual locations of the network points better than the conventional methods. There were no significant differences between RMSDs for all methods considered for the estimated heights of points 101 and 102. For point 104, the conventional methods were more accurate. This was due to the small simulated displacements of points 101 and 102.

### 3.2. Simulated Horizontal Network

**Figure 6:**
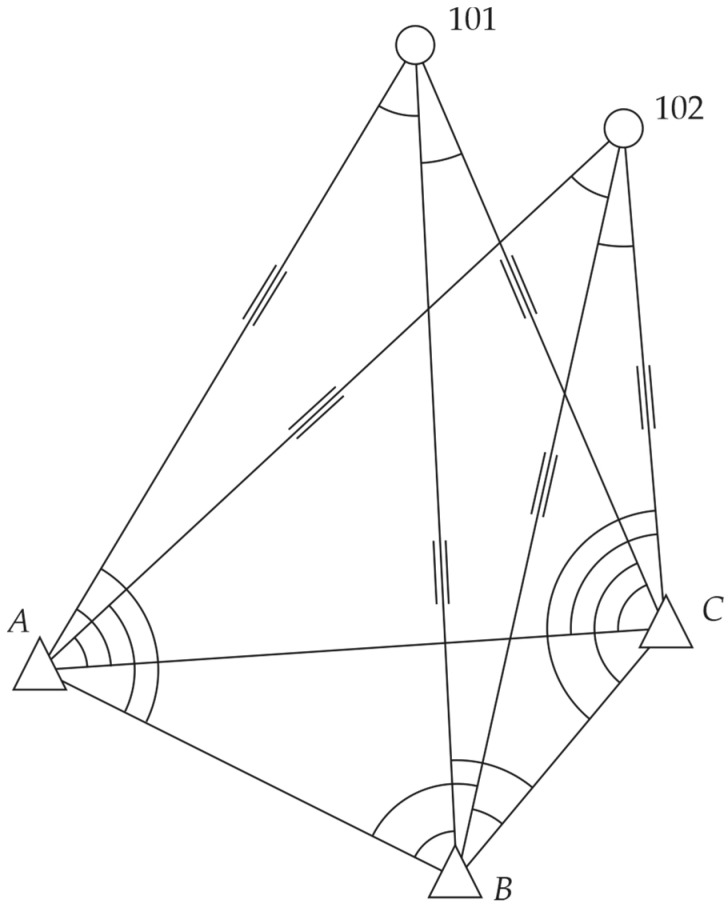
Simulated horizontal network.

The second numerical example concerned the simulated horizontal network established for deformation analysis. The simulated horizontal network is presented in Figure 6. It consisted of three reference points, *A* $\left(X_{A}=100\;m,\;Y_{A}=100\;m\right),$ *B* $\left(X_{B}=50\;m,\;Y_{B}=200\;m\right),$ and *C* $\left(X_{C}=110\;m,\;Y_{C}=250\;m\right)$, and two object points, 101 and 102 [10]. The observation vector included independent observations: 12 distance measurements (each distance was measured twice) and 16 horizontal angles at the measurement epoch. The assumed accuracies of the observations were 0.002 m and 0.0020 g, respectively; hence, the weight matrix was $P=0.002^{-2}\cdot I$ (for the LS method or M- and M_split_ estimation, one can assume P=I).

We let object point 101 be displaced during the measurements and assumed that the change of the coordinates *X*_101_ and *Y*_101_ of the object point 101 equaled 40 mm and 20 mm, respectively. These values corresponded to the coordinate differences between two measurement epochs in a previous paper [10]. The set consisted of two observation subsets (the measurements before and after the point displacement). The theoretical values of the observations of the first pseudo epoch were computed using the coordinates of three reference points and the theoretical coordinates of the object points 101 $\left(X_{101}=250\;m,\;Y_{101}=190\;m\right)$ and 102 $\left(X_{102}=230\;m,\;Y_{102}=240\;m\right)$. In contrast, the theoretical observations of the second pseudo epoch were computed by taking into account the shift of point 101 $\left(X_{101}=250,040\;m,\;Y_{101}=190,020\;m\right)$.

Like in the previous example, we considered two variants that differed from each other in the number of observations in both pseudo epochs. In Variant I, the distances $d_{101-B}$ and $d_{101-C}$ and horizontal angles $\alpha_{101-B-C}$ and $\alpha_{B-C-101}$ were measured after point 101 displacement. In Variant II, the distances $d_{101-B}$ and $d_{101-C}$ and horizontal angles $\alpha_{A-B-101}$, $\alpha_{101-B-C}$, $\alpha_{B-C-101}$, and $\alpha_{A-C-101}$ were measured after point 101 displacement.

Assuming that the observations were normally distributed, we simulated the observation set 5000 times in Mathcad 15.0. We also compared the following estimation methods: LS estimation, the Huber method with the steering parameter c=2, HLW estimation, and the two variants of M_split_ estimation (SMS and AMS methods). The starting vector $X_{\left(1\right)}^{0}$ of the iterative processes of M_split_ estimation of (11) and (16) was computed based on the coordinates of the reference points, “raw” measurements $d_{A-101}$, $d_{A-102}$, $\alpha_{101-A-C}$, and $\alpha_{102-A-C}$ and the simple trigonometric functions as proposed in [24]. The starting vector $X_{\left(2\right)}^{0}$ (applied in AMS estimation) was computed by adding 0.040 m to each element of the vector $X_{\left(1\right)}^{0}$.

During the estimation process, the increments $dX_{101}$, $dY_{101}$, $dX_{102}$, and $dY_{102}$ were calculated. As only point 101 was displaced during the measurements, we have presented only results for that point.

**Figure 7:**
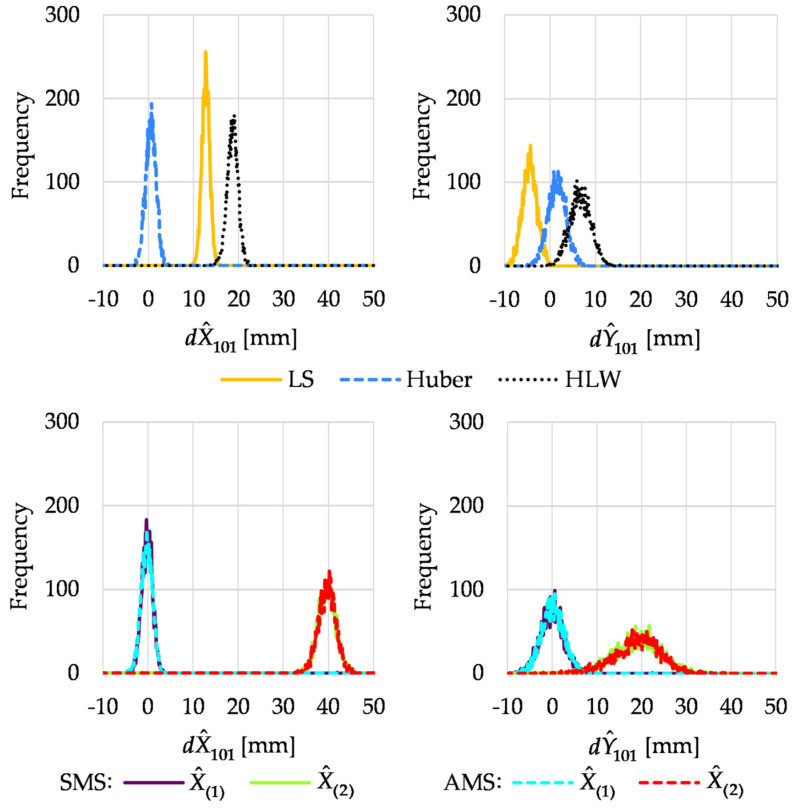
Histograms of estimated increments to point 101 coordinates in Variant I.

The histograms of $d{\hat{X}}_{101}$ and $d{\hat{Y}}_{101}$ obtained in Variant I are given in Figure 7.

Although only four observations were made after point 101 displacement in this variant, both variants of M_split_ estimation correctly indicated the shift. However, the histograms of $d{\hat{Y}}_{101}$ for the second solutions ${\hat{X}}_{\left(2\right)}$ were broad; their modes were close to 20 mm. The conventional methods failed except for the Huber method, for which the estimator histograms were pretty well located (nevertheless, the histogram of $d{\hat{Y}}_{101}$ was slightly shifted to the right).

**Figure 8:**
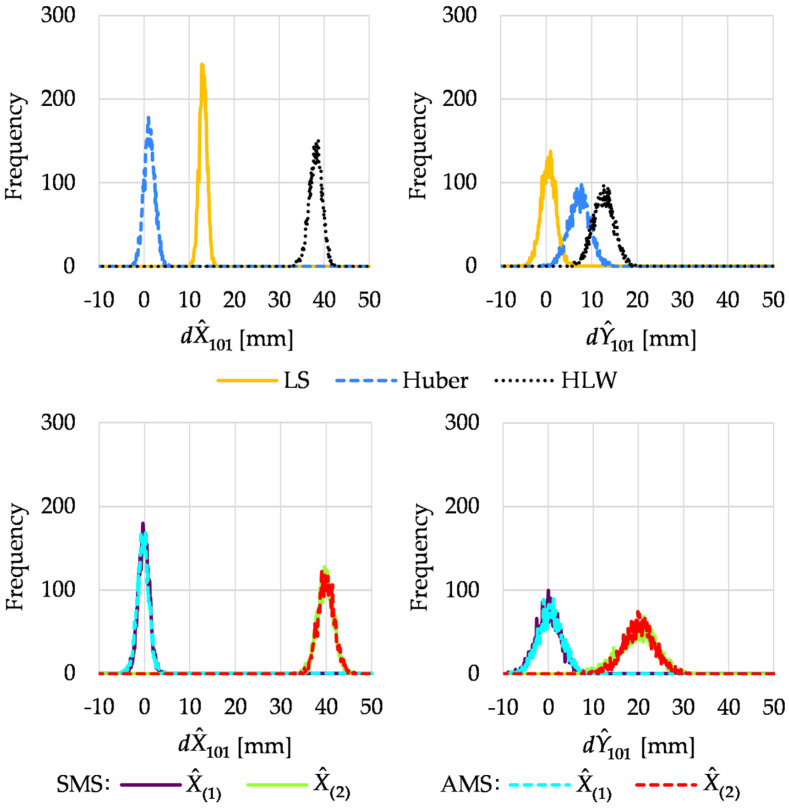
Histograms of estimated increments to point 101 coordinates in Variant II.

In Variant II, two more measurements were carried out after the point displacements. Histograms of estimated increments to point 101 coordinates are shown in Figure 8.

The results showed that all conventional methods did not deal with the higher number of outliers. The histograms indicated that the methods could not “tell” the regular observations from the outliers. Once again, the histograms showed that M_split_ estimation distinguished between the two pseudo epochs, and the results obtained corresponded to point 101 displacement.

As outlined in Section 3.1, we computed some descriptive statistics from the crude Monte Carlo simulations. Table 3 and Table 4 present the medians and RMSDs of the estimates of increments to point 101 coordinates. In Variant I, the medians and RMSDs confirmed the conclusions from the simple analysis of the histogram locations. The Huber method gave entirely satisfactory results, which meant that the four observations carried out after point 101 displacements were regarded as outliers, and their influence on the estimation results was significantly reduced. RMSDs obtained for the Huber method were like those of the M_split_ estimation variants (the solution ${\hat{X}}_{\left(1\right)}$). The medians showed that M_split_ estimation correctly assessed the point displacements. Thus, M_split_ estimation distinguished the first pseudo epoch from the second one.

The statistics obtained in Variant II confirmed the conclusions concerning the conventional methods. In many cases, they did not deal with the existence of two pseudo epochs. The results for both variants of M_split_ estimations were similar and slightly better than in Variant I. The medians for M_split_ estimation showed that the point shift was adequately assessed. This was due to the fact that more observations were carried out after the displacement of point 101; hence, it was easier for M_split_ estimation to correctly assign measurements to two pseudo epochs.

## 4. Discussion

The empirical tests showed the main differences between applying the conventional methods and M_split_ estimation when object points might move during measurements within one epoch. Loss or preservation of information about point displacements is essential. The examples presented in this paper prove that conventional methods may not manage point displacement during measurements. When applying robust M-estimation, observations from one pseudo epoch (before or after point displacements) should be assumed to be outliers. The robust methods can provide correct results only when the number of outlying observations (usually the group of observations performed after the point displacements) is small enough. We would surely lose information about point displacements by “ignoring” such a group of observations. We would also not know if the estimation results related to the first or the second pseudo epoch. By applying M_split_ estimation, one can preserve the information about point displacements and keep track of point movements during the measurement epoch. M_split_ estimation is used to determine two competitive versions of the parameters, such as the two versions of the object point coordinates (before and after the point displacement) in this case. In the given examples, both variants of M_split_ estimation distinguished the two groups of observations. Moreover, they correctly assessed the point displacements between the pseudo epochs. Comparing the results obtained for the M_split_ estimation variants, one can suggest applying the AMS method rather than SMS estimation for the problem addressed in this paper.

Robust estimation methods are not the only way to deal with outlying observations. Another option is statistical testing in the global or local case [41,42], which is supposed to indicate outlying observations. The flagged observations should be rejected from the observation set, hence eliminating their influence on the results. The procedure of statistical testing is also performed in deformation analysis. However, distinguishing outliers from displacements might be a problem [13]. It is also important to realize that there is no universal test for detecting outliers and, in fact, no rigorous tests for multiple outlier detection in least squares estimation [43]. Considering the problem of pseudo epochs, one should almost always expect that there is more than one observation regarded as an outlier (related to the pseudo epoch for which the number of observations is lower); hence, the application of statistical test might fail. On the other hand, even if the outliers are flagged correctly, one is losing information about the point movements during measurements, contrary to the application of M_split_ estimation. Talking about statistical procedures in deformation analysis, one should also mention testing the statistical significance of displacements, namely, CT (applying $\chi^{2}$ distribution), FT (involving Fisher–Snedecor distribution), or GCT (global congruence test) [14,44,45,46]. Undoubtedly, undiscovered point displacements will surely disturb all procedures mentioned. In further studies, it would be interesting to compare the proposed method to the statistical testing mentioned. We would also consider heterogeneous cases where observations have different accuracies and/or are correlated.

The efficacy of robust estimation or statistical tests for data cleaning is also problematic when the magnitude of point displacements during measurements is similar to the observation accuracies. Variant IV in the first test showed that M_split_ estimation was able to detect the displacements of such a magnitude only when the number of observations in both pseudo epochs was high enough.

The approach presented in this paper concerns point movements that are sudden and happen once in the epoch. If there are more such movements, one should consider M_split(*q*)_ estimation, where *q* is the number of expected pseudo epochs. The squared M_split(*q*)_ estimation was presented in [33] and the absolute M_split(3)_ estimation in [25]. Such an application would require further empirical analysis, especially for efficacy related to the minimal number of observations in each pseudo epoch. Another problem is when a point (or points) moves continuously during measurements. In this case, the approach based on the pseudo epoch seems problematic, and deformation analysis would probably require permanent measurements and other estimation methods [47,48,49].

## 5. Conclusions

If they occur, the displacements of object points during one measurement epoch stay undetected at the stage of measurements. Sudden and discrete point movements might mainly concern large networks where the number of observations is high; hence, a longer time is required for measurements. Pseudo epochs might also occur in networks established to determine terrain surface deformations resulting, for example, from uplifts or mining damages, where the vertical displacements might be sudden and have a relatively high magnitude. Pseudo epoch analysis based on M_split_ estimation application seems reasonable and advisable in such networks or objects of such characteristics. The efficacy of the proposed procedure depends on the magnitude of displacements and the number of observations in each pseudo epoch (the higher the displacement magnitude and the more similar the numbers of pseudo epoch observations, the more reliable the results are expected to be).

Deformation analysis is a complicated procedure that is sensitive to unexpected and undetectable disturbances, such as outliers, instability of possible reference points, etc. Thus, procedures for detecting outliers or checking the stability of reference points are always used. For the same reasons, one might apply the procedure proposed in this paper to check object point movements during measurements and assess the displacement values.

## Figures and Tables

**Table 1:** Medians (mm) of the chosen object point height estimates from simulations.

Variant	Point	LS	Huber	HLW	$\mathrm{SMS}\;{\hat{X}}_{\left(1\right)}$	$\mathrm{SMS}\;{\hat{X}}_{\left(2\right)}$	$\mathrm{AMS}\;{\hat{X}}_{\left(1\right)}$	$\mathrm{AMS}\;{\hat{X}}_{\left(2\right)}$
Variant I	101	2.0	0.0	4.1	−0.1	10.1	−0.1	10.0
	102	4.1	0.1	0.9	−0.1	19.9	0.0	20.2
	104	1.3	0.0	0.2	0.0	3.0	−0.1	1.0
Variant II	101	6.8	6.8	9.1	0.9	10.1	0.4	10.1
	102	11.4	11.6	10.6	0.9	20.0	0.3	20.0
	104	1.8	1.8	0.2	0.9	0.0	0.3	0.0
Variant III	101	0.8	0.6	1.0	0.9	1.6	0.4	1.9
	102	0.3	0.3	0.4	−0.1	1.5	−0.1	1.4
	104	0.2	0.1	0.1	−0.2	0.8	−0.1	0.7
Variant IV	101	1.6	1.6	1.8	0.8	2.5	1.0	2.4
	102	1.1	1.1	1.4	0.3	2.1	0.4	2.1
	104	0.3	0.3	0.1	0.2	0.2	0.2	0.1

**Table 2:** RMSDs (mm) of the chosen object point height estimates from simulations.

Variant	Point	LS	Huber	HLW	$\mathrm{SMS}\;{\hat{X}}_{\left(1\right)}$	$\mathrm{SMS}\;{\hat{X}}_{\left(2\right)}$	$\mathrm{AMS}\;{\hat{X}}_{\left(1\right)}$	$\mathrm{AMS}\;{\hat{X}}_{\left(2\right)}$
Variant I	101	2.1	0.6	4.1	1.2	1.4	1.2	1.9
	102	4.1	0.5	1.0	0.9	1.0	0.9	1.0
	104	1.4	0.4	0.4	1.0	5.1	1.0	4.7
Variant II	101	6.9	6.8	9.1	3.9	1.2	2.7	1.7
	102	11.4	12.2	10.7	3.9	0.7	3.1	0.7
	104	1.9	1.8	0.4	3.9	1.4	2.1	2.7
Variant III	101	1.0	0.9	1.1	1.7	2.1	1.6	2.1
	102	0.5	0.5	0.6	0.8	1.6	0.8	1.6
	104	0.4	0.4	0.4	0.9	1.3	0.8	1.2
Variant IV	101	1.7	1.7	1.9	1.7	1.6	1.8	1.6
	102	1.2	1.2	1.5	1.0	1.0	0.9	1.0
	104	0.4	0.4	0.4	1.0	1.2	1.0	1.1

**Table 3:** Medians (mm) of the estimates of increments to point 101 coordinates from simulations.

Variant	Estimator	LS	Huber	HLW	$\mathrm{SMS}\;{\hat{X}}_{\left(1\right)}$	$\mathrm{SMS}\;{\hat{X}}_{\left(2\right)}$	$\mathrm{AMS}\;{\hat{X}}_{\left(1\right)}$	$\mathrm{AMS}\;{\hat{X}}_{\left(2\right)}$
Variant I	$d{\hat{X}}_{101}$	−4.4	0.6	18.8	−0.1	39.8	−0.2	39.9
	$d{\hat{Y}}_{101}$	0.0	1.6	6.6	0.0	19.2	0.2	19.4
Variant II	$d{\hat{X}}_{101}$	13.2	1.2	38.2	−0.1	40.0	−0.3	40.0
	$d{\hat{Y}}_{101}$	0.6	7.5	12.6	0.1	20.1	0.5	20.2

**Table 4:** RMSDs (mm) of the estimates of increments to point 101 coordinates from simulations.

Variant	Estimator	LS	Huber	HLW	$\mathrm{SMS}\;{\hat{X}}_{\left(1\right)}$	$\mathrm{SMS}\;{\hat{X}}_{\left(2\right)}$	$\mathrm{AMS}\;{\hat{X}}_{\left(1\right)}$	$\mathrm{AMS}\;{\hat{X}}_{\left(2\right)}$
Variant I	$d{\hat{X}}_{101}$	12.8	1.3	18.8	1.3	2.0	1.3	2.0
	$d{\hat{Y}}_{101}$	4.7	2.5	7.1	2.6	5.3	2.5	5.4
Variant II	$d{\hat{X}}_{101}$	13.2	1.7	38.2	1.2	1.8	1.3	1.8
	$d{\hat{Y}}_{101}$	1.7	7.8	12.8	2.7	4.1	2.8	3.5

## Data Availability

The data presented in this study are available on request from the corresponding author.
